# A novel highly quantitative and reproducible assay for the detection of anti-SARS-CoV-2 IgG and IgM antibodies

**DOI:** 10.1038/s41598-021-84387-3

**Published:** 2021-03-04

**Authors:** Kenta Noda, Kouki Matsuda, Shigehiro Yagishita, Kenji Maeda, Yutaro Akiyama, Junko Terada-Hirashima, Hiromichi Matsushita, Satoshi Iwata, Kazuto Yamashita, Yusuke Atarashi, Shunsuke Watanabe, Nobuyuki Ide, Tomokazu Yoshida, Norio Ohmagari, Hiroaki Mitsuya, Akinobu Hamada

**Affiliations:** 1 Central Research Laboratories, Sysmex Corporation, Kobe, Hyogo Japan; 2 Department of Refractory Viral Infections, National Center for Global Health and Medicine (NCGM) Research Institute, Tokyo, Japan; 3 Division of Molecular Pharmacology, National Cancer Center Research Institute, Tokyo, Japan; 4 Disease Control and Prevention Center (DCC), NCGM, Tokyo, Japan; 5 Center for Clinical Science, NCGM, Tokyo, Japan; 6 Respiratory Medicine, NCGM Center Hospital, Tokyo, Japan; 7 Department of Laboratory Medicine, National Cancer Center Hospital, Tokyo, Japan; 8 Department of Infectious Diseases, National Cancer Center Hospital, Tokyo, Japan; 9 Bio-Diagnostic Reagent Technology Center, Sysmex Corporation, Kobe, Hyogo Japan; 10 Experimental Retrovirology Section, HIV and AIDS Malignancy Branch, National Cancer Institute, National Institutes of Health, Bethesda, MD USA; 11 Department of Clinical Sciences, Kumamoto University Hospital, Kumamoto, Japan

**Keywords:** Analytical biochemistry, Immunological techniques, Infectious-disease diagnostics

## Abstract

The quantitative range and reproducibility of current serological tests for severe acute respiratory syndrome coronavirus-2 (SARS-CoV-2) are not optimized. Herein, we developed a diagnostic test that detects SARS-CoV-2 IgG and IgM with high quantitativeness and reproducibility and low interference. The system was based on the high-sensitivity chemiluminescence enzyme immunoassay (HISCL) platform and detects IgG and IgM specific to SARS-CoV-2 spike and nucleocapsid proteins. Quantification accuracy and reproducibility were evaluated using serially diluted samples from 60 SARS-CoV-2-infected patients. Assay performance was evaluated using serum samples from the SARS-CoV-2-infected patients and 500 SARS-CoV-2-negative serum samples collected before the emergence of SARS-CoV-2. The system showed high quantification accuracy (range, 10^2^), high reproducibility (within 5%), and no cross-reaction between SARS1- and MERS-S proteins. Detection accuracy was 98.3% and 93.3% for IgG and IgM against spike proteins and 100% and 71.7% for IgG and IgM against nucleocapsid proteins, respectively. Mean antibody levels were > 10 times that in negative samples upon admission and > 100 times that at convalescent periods. Clinical severity upon admission was not correlated with IgG or IgM levels. This highly quantitative, reproducible assay system with high clinical performance may help analyze temporal serological/immunological profiles of SARS-CoV-2 infection and SARS-CoV-2 vaccine effectiveness.

## Introduction

The epidemic triggered by the severe acute respiratory syndrome coronavirus 2 (SARS-CoV-2) that originated in China has rapidly spread worldwide. SARS-CoV-2 causes severe, acute—and in some cases—fatal coronavirus disease in humans, named COVID-19, and is considered a global public threat^1–4^. However, no specific therapeutic agents for COVID-19 are currently available. Moreover, SARS-CoV-2 may persist in some convalescent COVID-19 survivors, and its infection could continue to recur, causing a continued pandemic. In such circumstances, developing a high-performance and cost-effective diagnostic tool for COVID-19 is a high priority.

Currently, polymerase chain reaction (PCR) testing based on the detection of the SARS-CoV-2 genome has been widely employed in clinical settings and used as a gold-standard to confirm positive and negative infections^5,6^. More recently, antigen testing has also been used, although it is slightly less sensitive and precise than PCR testing^7^.

Antibody testing aimed at detecting SARS-CoV-2-related immunity of patients is believed to be associated with the clinical history of the infected patients and their virus-neutralizing immune response^8^. Blood-based antibody diagnostic analysis commonly assesses IgG and IgM titers^9–11^. It has been reported that IgM levels increase early after infection in common viral infections, as well as in COVID-19, followed by an increase in IgG levels^12^. However, it has been reported that in the early stage of SARS-CoV-2 infection IgG levels increase rather than IgM levels^13,14^. Additionally, several methods have been developed for measuring the titers of antibodies against SARS-CoV-2 proteins, such as nucleocapsid proteins and receptor proteins (Spike protein, S1 domain, and receptor binding domain)^15–19^. Because the relationship between antibody levels and clinical response is still unclear^20–22^, it is necessary to identify the SARS-CoV-2 protein, which can be used as a target in diagnostic tests with a better diagnostic performance. Recently, some antibody-test kits for SARS-CoV-2 have been made available for research; however, their performance is poor, and they generate unreliable and low-quality results^17,23–25^. Currently available immunochromatographic COVID-19 antibody testing gives rise to qualitative detection; thus accounting for false-negative results in clinical practice. Although there are quantitative detection kits using ELISA for research purposes, the measurement accuracy and range are limited. In addition, commercially available detection reagents that are used for an automatic immunoassay instrument are only qualitative determinations^26,27^. In future, a high-precision quantitative assay is required not only for the purpose of positive qualitative determination but also for monitoring the antibody titer of vaccine administration and setting a threshold value. Additionally, to observe antibody titers over time, a monitoring system that is quantitative and has a wide measurement range is required. Therefore, a high-quality serological test with appropriate analytical standards that is available at a reasonable cost is warranted.

This study describes a novel quantitative assay using a fully automated immunochemistry analyzer that employs chemiluminescence enzyme immunoassay (CLEIA) methodology^28,29^, HISCL (Sysmex Corporation, Kobe, Japan), to simultaneously detect IgG and IgM antibodies against two SARS-CoV-2 antigens, the spike (S) protein and the nucleocapsid (N) protein (N-IgG, S-IgG, N-IgM, and S-IgM). HISCL is widely used in several clinical fields due to its rapid reaction (17 min), wide dynamic ranges, and high reproducibility compared with standard enzyme-linked immunosorbent assay (ELISA). The analytical performance of the HISCL-based serological assay was evaluated with respect to its sensitivity, specificity, and reproducibility. Moreover, given the clinical nature of this pilot study, the levels of N-IgG, S-IgG, N-IgM, and S-IgM in SARS-CoV-2-infected patients at the time of hospital admission and during convalescence were also evaluated using the validated analytical method.

## Results

### Analytical performance

#### Development of an analytical method for detecting SARS CoV-2 antigen

##### Standard curve

**Figure 1:**
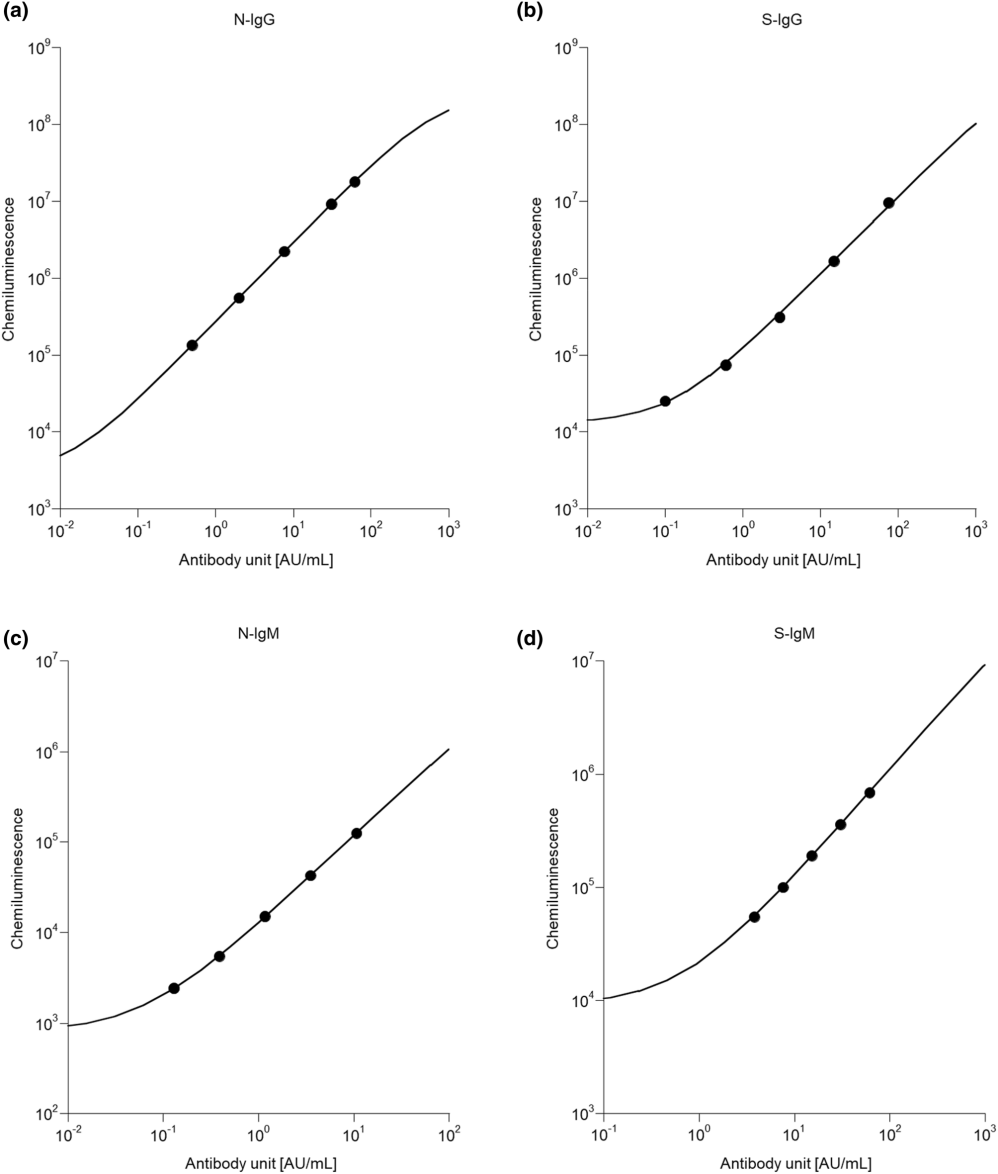
Relationship between chemiluminescence and the levels of each antibody. Working curve of N-IgG (**a**), S-IgG (**b**), N-IgM (**c**), and S-IgM (**d**).

A standard curve was plotted based on the chemiluminescence intensity of the diluted SARS-CoV-2-positive plasma to investigate the relationship between the emission intensity and the antibody concentration (Fig. 1).

##### Reproducibility

The precision of the assay was determined using two SARS-CoV-2 concentrations. Within-assay coefficient of variation (CV) was determined using 10 replicates for each sample. The within-run CVs for N-IgG, S-IgG, N-IgM, and S-IgM were less than 2.1%, 3.3%, 2.1%, and 1.2%, respectively (Supplemental Table 1).

##### Interferences

The interference of common blood components was assessed by adding interfering substances into plasma samples. The changes between samples with and without all types of interfering substances were less than 15%.

##### Cross-reactivity

In the assay using the nucleocapsid protein, the addition of the SARS-CoV-S antigen resulted in the same level of inhibition in all positive specimens as the addition of the SARS-CoV-2 antigen. The inhibition rates of the NL63 and 229E antigens were 40–70% higher than those of the SARS-CoV-2 antigens, depending on the specimens. Furthermore, in the assay using the S protein, little cross-reactivity was observed in all specimens (Supplemental Figure 1). When the antigen was added to the negative specimens, the quantitative values were all less than 0.5 U/mL.

### Clinical performance

#### Sensitivity, specificity, and AUC assessment

**Figure 2:**
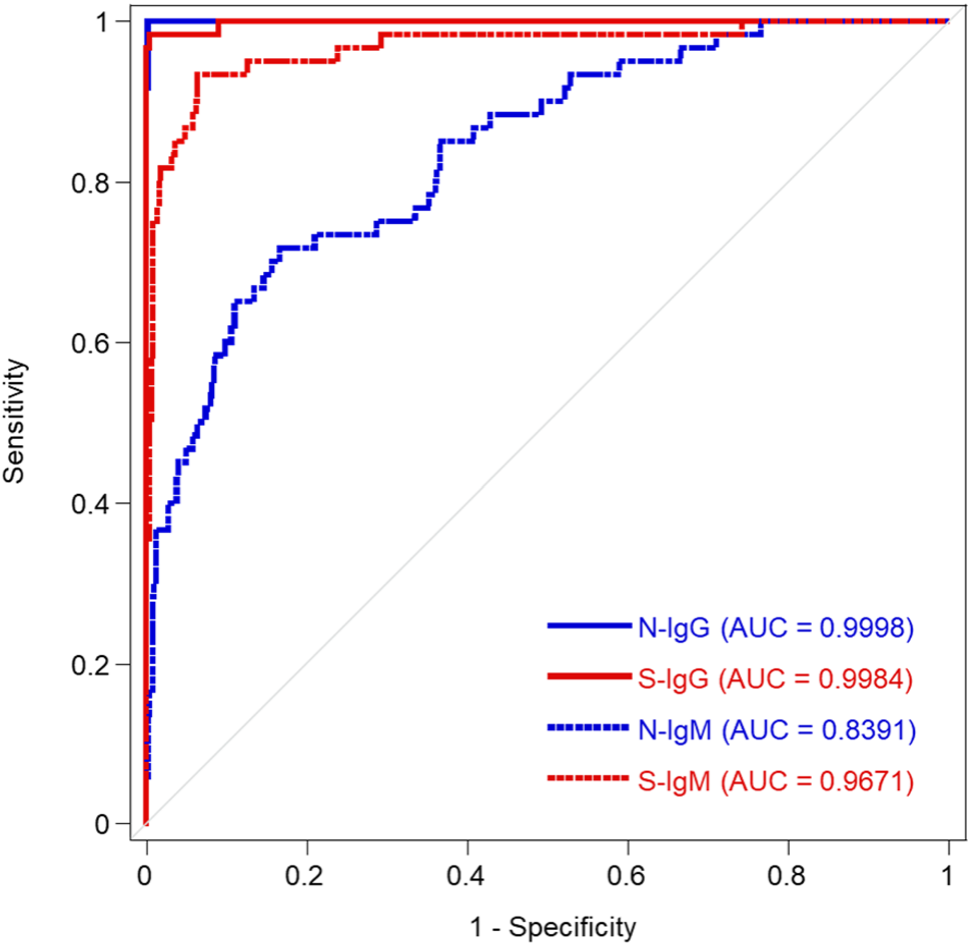
Clinical performance of SARS-CoV-2 antibodies. Receiver operating characteristic (ROC) curves for convalescent and negative samples. Blue line, N-IgG; red line, S-IgG; blue dotted line, N-IgM; red dotted line, S-IgM estimated by the logistic regression model. Thin gray line represents the random classification.

The ROC curves for N-IgG, S-IgG, N-IgM, and S-IgM in the positive and negative subjects are shown in Fig. 2. The AUC for N-IgG, S-IgG, N-IgM, and S-IgM were 0.9998, 0.9984, 0.8391, and 0.9671, respectively. The sensitivity and specificity of N-IgG and S-IgG were over 98%. The sensitivity and specificity of N-IgM were 71.7% and 83.4%, whereas that for S-IgM were 93.3% and 93.6%, respectively (Supplemental Table 1).

**Figure 3:**
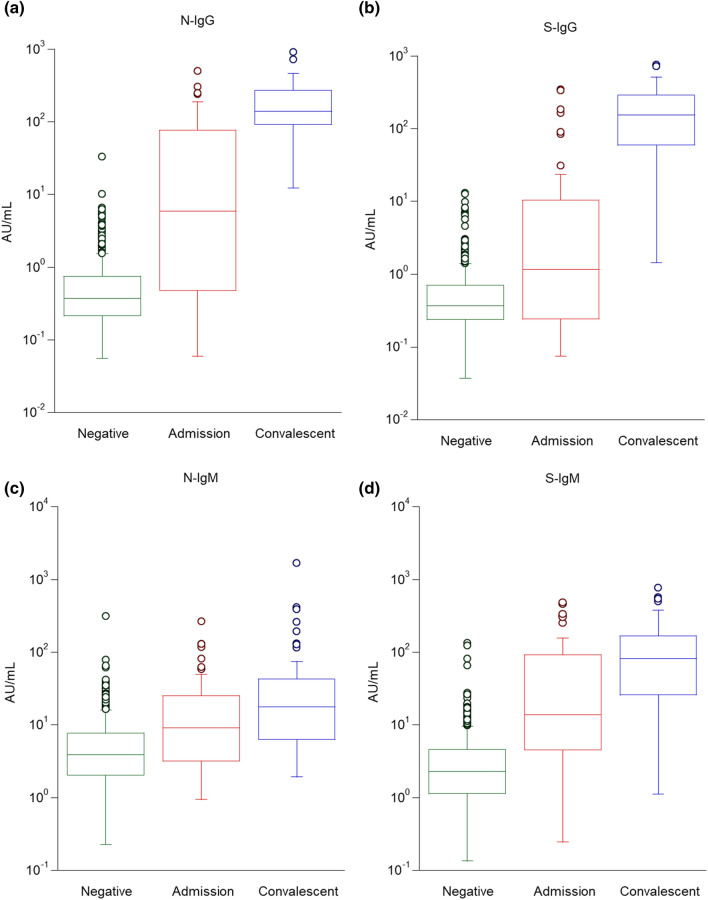
Relationship between antibody levels at negative, admission, and convalescent patients. A total of 500 negative samples, 50 admission samples, and 60 convalescent samples were analyzed using each antibody detection reagent. N-IgG (**a**), S-IgG (**b**), N-IgM (**c**), and S-IgM (**d**) levels.

The number of SARS-CoV-2 binding antibodies (IgG and IgM) was evaluated in all patients at the time of admission as well as during convalescence. The IgG and IgM levels in blood samples from 500 non-infected patients were also assessed as a negative control. As shown in Fig. 3, the antibody amount in the serum samples from convalescent patients was higher than that at the time of admission for all tested antibodies (N-IgG, S-IgG, N-IgM, and S-IgM). Overall, the on-admission N-IgM and S-IgM values were higher than N-IgG and S-IgG (Table 1), which was in agreement with the general understanding that the levels of IgM increase more rapidly than those of IgG after antigen exposure in patients with a viral infection. However, even though the levels of N-IgM and S-IgM were higher in patients with COVID-19, this is not a practical approach for the diagnosis of COVID-19 as the blood samples from healthy donors exhibit a high rate of false positives.

**Table 1:** Changes in IgG and IgM antibodies in response to the presence of SARS-CoV-2 antigen during hospital admission and convalescence.

Antibodies	Admission				Convalescent				*P-*value: admission vs convalescent		
	Moderate	Severe	Critical	*P*-value	Moderate	Severe	Critical	*P-*value	Moderate	Severe	Critical
N-IgG	3.4	6.6	51.5	M vs S, n.s.	101.8	214.7	159.2	M vs S, < 0.01	< 0.001	< 0.001	n.s.
(AU/mL)	(0.4–72.8)	(1.6–78.8)	(2.1–156.4)	S vs C, n.s.	(62.4–205.3)	(127.2–345.8)	(132.2–282.6)	S vs C, n.s.			
				M vs C, n.s.				M vs C, n.s.			
S-IgG	1.6	0.5	6.3	M vs S, n.s.	93	242.6	216.4	M vs S, < 0.01	< 0.001	< 0.001	< 0.05
(AU/mL)	(0.2–15.8)	(0.3–2.3)	(1.1–104.5)	S vs C, n.s.	(44.6–217.0)	(154.9–375.9)	(123.6–448.7)	S vs C, n.s.			
				M vs C, n.s.				M vs C, n.s.			
N-IgM	10.4	7.6	15.2	M vs S, n.s.	13.9	26.5	18.6	M vs S, n.s.	n.s.	< 0.05	n.s.
(AU/mL)	(2.7–19.3)	(3.5–59.5)	(9.4–28.4)	S vs C, n.s.	(5.5–40.5)	(15.0–49.6)	(16.6–28.7)	S vs C, n.s.			
				M vs C, n.s.				M vs C, n.s.			
S-IgM	22.3	9.4	125.8	M vs S, n.s.	51.1	98	196.1	M vs S, n.s.	n.s.	< 0.001	n.s.
(AU/mL)	(3.6–111.1)	(3.5–37.5)	(41.7–304.5)	S vs C, < 0.05	(15.6–153.7)	(57.4–166.3)	(59.3–607.6)	S vs C, n.s.			
				M vs C, n.s.				M vs C, n.s.			

Antibody concentrations are expressed as medians (distribution range). *P*-values were obtained by Mann–Whitney *U* test and Steel–Dwass test. *C* critical, *M* moderate, *S* severe, *n.s.* not significant.

Supplemental Figure 2 shows a comparison between the HISCL analysis system and the Euroimmun SARS-CoV-2 IgG ELISA kit available for research purposes. A total of 19 cases between "on admission" and "convalescence" were evaluated using both the assays, and both revealed significant differences between the admission and convalescence samples (Supplemental Figure 2). Nevertheless, the HISCL system seemed to be better at detecting the differences between the two time points.

Clinical information for all patients was obtained and analyzed (Table 2). All patients were divided into three groups (Moderate, Severe, and Critical) based on the symptoms and treatments during the disease course (for definition, see the footnote in Table 2). Next, it was investigated whether the severity of each patient could influence the antibody level. Regarding the titer of antibodies at the time of admission, patients in critical condition exhibited the highest levels of all four antibodies among the three groups; however, the difference was significant (*P* < 0.05) only for S-IgM (Table 1). Additionally, during convalescence, patients with severe/critical disease course exhibited higher antibody levels than those with moderate disease. This result may suggest that the duration of exposure to high titers of a virus (in patients in severe and critical conditions) is an important factor for acquiring potent immunity against SARS-CoV-2.

**Table 2:** Patients and sample collection information.

Variables	Early stage	Convalescent
Sample collection date	From 2/24/2020 to 5/3/2020	From 4/11/2020 to 6/2/2020
Total number	50	60
Sex (M/F)	39 / 11	48 / 12
Age, median (range)	50 (23–85)	53 (25–85)
Days since symptoms, median (range)	9 (1–25)	36 (20–95)
Disease severity^1^ (moderate/severe/critical)	26/19/5	36/19/5

^1^Disease severity definitions—Moderate: febrile or fatigue, and with/without pneumonia, and no oxygen inhalation required; Severe: febrile, fatigue, dyspnea, severe pneumonia identified, and oxygen inhalation required; Critical: febrile, fatigue, severe dyspnea, critical pneumonia identified, and positive pressure ventilation plus extracorporeal membrane oxygenation (ECMO) required.

## Discussion

Serological testing to detect anti-SARS-CoV-2‒specific antibodies is an important approach to understand the extent of COVID-19 spread in the community. These specific antibodies are immunological evidence of exposure to the virus. SARS-CoV-2-triggered immunity is characterized by an early increase in IgM levels, followed by an increase in IgG levels in the first few days post-infection. Subsequently, these antibodies are immunological biomarkers that indicate the infection experience, even after the virus has been eliminated. Immunological chromatography has been used as a qualitative test to distinguish between positive and negative results. However, there is a need for a rapid and easy method that can quantitatively measure the antibody levels in the blood. This study presents a highly sensitive quantitative test that can not only detect SARS-CoV-2 infection but also quantifies antibody levels that define "acquired immunity".

The method developed herein aimed to detect IgG and IgM antibodies against the SARS-CoV-2 receptor-binding domain (RBD). In particular, quantification of the S and N proteins and evaluating the levels of antibodies associated with acquired immunity, are expected to be useful indicators for the development of a vaccine. This current assay qualification builds on previously described ELISAs^30^ that focused on capturing antibodies against the RBD, due to its critical role as the primary target of neutralizing antibodies^31^. Additionally, unlike the trimeric stabilized S protein, which is more challenging to produce, RBD is a highly stable structure that can be produced and scaled-up for large-scale ELISA testing. Given the immunogenic nature of the RBD, it likely represents a reasonable target to quantify population-level exposure and define antibody levels, which may ultimately be associated with immunity. However, additional viral components, including the membrane protein‒the N or envelope protein—that are generated in large amounts during infection^32,33^ could provide enhanced diagnostic value. This is of particular interest during early infection, when antigen concentration may influence the kinetics of antibody evolution.

Furthermore, quantitative testing could help diagnose the infection at its early stages, as antigen concentrations may affect antibody production. Besides, the quantification of antibody levels and the immune response can enhance our understanding of SARS-CoV-2. Although some antibody testing methods have been reported, it is unclear whether the results of qualitative antibody tests reflect immunity against SARS-CoV-2. Moreover, antibodies that target alternate sites on the spike antigen or with the ability to drive additional antibody effector functions may also contribute to immunity. Thus, next-generation assay development, using alternate antigens, may provide early diagnostic value in the absence of RNA testing and might provide additional insights into antibody levels that reflect the immune response.

Herein, we described a rapid and high-reproducible diagnostic system for N-IgG, S-IgG, N-IgM, and S-IgM using the HISCL platform. The developed assay exhibited good reproducibility and a wide dynamic range. Furthermore, the antibody levels measured using these assays were not influenced by standard blood components, and the specimens harboring coronavirus protein showed the highest antibody titer. As a result, the S protein assay specifically detected the SARS-CoV-2 S protein but did not detect antibodies that targeted the proteins of the common cold coronavirus (Supplemental Figure 1). To avoid potential cross reactivity of S protein-targeting antibodies, the new system was designed based on the recognition of the three-dimensional structure of the targeted antigen^34^. In contrast, assays targeting the N protein may also detect proteins of the cold coronavirus, probably due to the high homology of the N proteins among the coronavirus family^35,36^. However, COVID-19 is clinically different from the common cold coronavirus-infection^37^, thus cross-reactivity with SARS-CoV-2 S protein might be considered acceptable for the diagnosis of COVID-19. Therefore, the analytical performance of the proposed novel antibody assay has sufficient for clinical application.

Although IgG antibody tests have recently become available as unapproved diagnostic kits, many have poor performance as clinical diagnostic tools. The strategy of using the HISCL analysis system can serve as a robust approach to quantify the levels of IgG and IgM antibodies against SARS-CoV-2. To fully explore the potential of the new antibody assay, its detection results were compared with those obtained with commercially available ELISA. This improved detection capacity may be attributed to the wider dynamic range of the HISCL-based analysis system compared to that of reference ELISA assay, which is dependent on the absorbance, OD ratio value, and detection system. These results demonstrate the superiority of the HISCL method for measuring a wide range of antibody titers.

**Figure 4:**
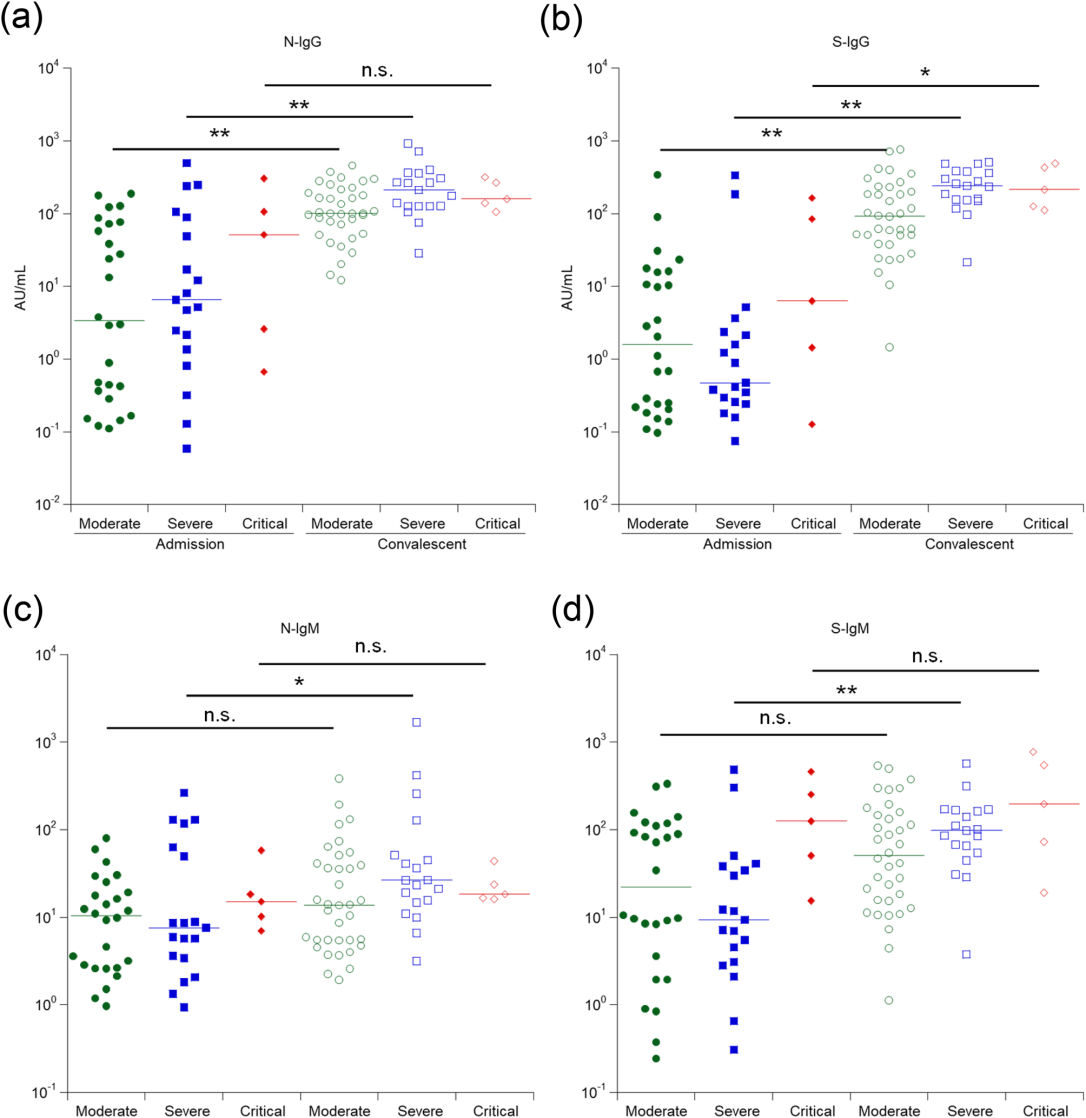
Relationship between severity status and antibody levels. Comparison of antibody titers on admission and convalescent for N-IgG (**a**), S-IgG (**b**), N-IgM (**c**), and S-IgM (**d**). *P > 0.05, **P > 0.01.

Maeda et al. confirmed that the concentration of neutralizing antibodies varies in patients, and the neutralizing activity and total antibody levels did not always correlate (manuscript in preparation). It is predicted that the SARS-CoV-2 RBD may be an important target for such neutralizing antibodies^38–41^. However, assessing these associations requires the accurate detection of the antibody levels at the following time points: at the beginning of the infection, at the start of treatment, during recovery, and after treatment. Herein, it was shown that is possible to detect some differences in the blood IgG levels among patients in the convalescent stage who exhibited moderate or severe/critical disease conditions (Fig. 4), suggesting the importance of a highly quantitative SARS-CoV-2 antibody detection system to accurately evaluate the medical status. Additionally, this study showed that the anti-SARS-CoV-2 IgM levels increase in the early phase of the disease in many patients; the earliest IgM detection was on day three after onset (data not shown). Indeed, significantly higher S-IgM levels were detected on admission in patients with the critical disease. To further explore these differences, interleukin (IL)-4 levels, which are considered to be important for the class-switching from IgM to IgG, were also examined, revealing that patients who later entered a critical condition had significantly higher IL-4 levels in the blood on admission (Supplemental Figure 3). Even though the mechanism underlying IL-4 increase in patients with critical COVID-19 course is unknown, the data suggest that the IL-4 levels combined with the anti-SARS-CoV-2-S-IgM data at the onset of the disease may help predict whether patients will enter a critical status in the future, requiring assisted ventilation or extracorporeal membrane oxygenation support. In addition, the collected data also suggest that highly sensitive anti-SARS-CoV-2 IgM detection may be useful for diagnosing and predicting disease severity.

There are a few limitations to this study. First, as the objective of the study was to establish a quantitative antibody test, only a few blood serum samples were obtained from patients infected with COVID-19, as well as data from patients who have been cured of the disease as stored samples were analyzed. Therefore, it is necessary to evaluate the medical implications of this examination method for prospective tests in the future. Furthermore, the change in antibody levels prior to and following vaccine administration is expected to be an important factor during analysis. As our quantitative antibody assay is the preferred method for monitoring the response of the immune system to COVID-19 following vaccination, the Japanese government is planning to begin vaccinations in March 2021. Thus, we are currently planning to conduct an observational study on the infection rate and severity in clinical practice by monitoring antibody levels in healthcare workers, office staff, and patients following vaccination.

In summary, we developed a novel anti-SARS-CoV-2 antibody detection system to accurately and robustly measure the levels of IgG and IgM antibodies in patients infected with SARS-CoV-2. This new system may play a valuable role in clinical practice and in understanding the ongoing COVID-19 epidemic.

## Methods

### Human clinical specimens

Sixty patients who were clinically diagnosed with COVID-19 and admitted to the National Center for Global Health and Medicine (NCGM) in Tokyo were enrolled in the study. All patients were confirmed to be SARS-CoV-2-positive using RNA-quantitative PCR on nasopharyngeal swab samples at the time of enrollment in the study (Table 2 and Supplementary Table 2). A total of 500 serum samples from cancer patients (337/163 male/female patients; age range: 7–87 years old) were provided by the National Cancer Center Biobank, Japan and used as the negative control samples. The Ethics Committee from the NCGM and the National Cancer Center approved this study (NCGM-G-003472-02, NCC 2020-026). Each patient provided written informed consent, and this study abided by the Declaration of Helsinki principles.

### Recombinant antigen production

The recombinant nucleocapsid proteins were produced based on the following accession number sequences: SARS-CoV-2, YP_009724397; SARS-CoV, YP_009825061; MERS-CoV, YP_009047211; HCoV-HKU1, YP_173242; HCoV-OC43, YP_009555245; HCoV-NL63, YP_003771; and HCoV-229E, NP_073556. The recombinant S1 regions of the spike proteins were produced based on the following accession number sequences: SARS-CoV-2, YP_009724390; SARS-CoV, YP_009825051; MERS-CoV, YP_009047204; HCoV-HKU1, YP_173238; HCoV-OC43, NP_937950; HCoV-NL63, YP_003767; and HCoV-229E, NP_073551. Each sequence was cloned into a pcDNA3.4 vector (Thermo Fisher Scientific, Waltham, MA, USA) with a C-terminus His-tag and transfected into Expi293 cells (Thermo Fisher Scientific) according to the manufacturer’s protocol. The supernatants were harvested 6 days post-transfection. Recombinant antigens were purified using a HisTrap HP column (Cytiva, Marlborough, MA, USA) and HiLoad 26/600 Superdex 200 pg column (Cytiva). Purified SARS-CoV-2 nucleocapsid protein and S1 protein were respectively coupled with magnetic beads using 1-ethyl-3-(3-dimethylaminopropyl)carbodiimide (Dojindo Molecular Technologies Inc., Kumamoto, Japan) and N-hydroxysuccinimide (Sigma-Aldrich, St. Louis, MO, USA).

### Assay description

HISCL anti-SARS-CoV-2 immunoassay was developed to quantify the titer of IgG and IgM antibodies in human serum or plasma. HISCL was operated in a fully automatic manner using the chemiluminescent sandwich principle. In this system, the serum or plasma sample first reacted with SARS-CoV-2-specific recombinant antigens bound to magnetic beads. After bound/free separation, the antigen–antibody complex was incubated with an alkaline phosphatase-conjugated antibody against human IgG or IgM to form a sandwich immunocomplex. After a second bound/free separation, a luminescent substrate was added into the solution to allow for luminescence measurement. Chemiluminescence intensity was acquired within 17 min following the addition of the substrate. The temperature of the reaction chamber was maintained at 42 °C throughout the procedure.

### Analytical performance

#### Calibrator and control preparation

Calibrators and controls were prepared using commercially available SARS-CoV-2 positive samples from Cantor Bioconnect (Toronto, Canada) and TRINA BIOREACTIVES (Naenikon, Switzerland). The calibrators were prepared by serial dilutions of SARS-CoV-2 positive samples with phosphate buffer. Each calibrator was measured three times. The assigned value of the calibrator was defined based on the cut-off values. The assay value of the antibody was calculated from the standard curve obtained from the logistic regression analysis.

#### Interferences

Potential interference materials were added to SARS-CoV-2 patient plasma up to the following concentrations: free-form bilirubin (up to 200 mg/L), conjugated-form bilirubin (up to 200 mg/L), chyle (up to 1600 FTU), hemoglobin (up to 500 mg/L), and rheumatoid factor (up to 4000 IU/L). These reagents were obtained from Interference Check A Plus and Interference Check Rheumatoid Factor (Sysmex Corporation).

#### Cross-reactivity

The specificity of the novel assay was evaluated by measuring samples with recombinant proteins from coronaviruses strains (SARS-CoV-2, SARS1, MERS, OC43, HKU1, 229E, NL63). Each antigen was added to the commercially available SARS-CoV-2 antibody-positive and negative samples so that the final concentration of the antigen in the measured sample was 20 µg/mL. Cross-reactivity was assessed based on the rate of inhibition by antigen addition.

### Clinical performance

#### Determination of sensitivity, specificity, and area under the curve (AUC)

To determine the AUC, sensitivity, and specificity, 500 negative and 60 positive serum samples were measured with each assay. AUC was determined by analyzing a receiver operating characteristic (ROC) curve. The sensitivity and specificity were calculated using a threshold point defined based on the Youden Index.

### Statistical analysis

The overall diagnostic accuracies of each antibody marker were evaluated by ROC analysis and non-parametric pairwise comparisons were evaluated by Mann–Whitney *U* test using StatFlex v.7.0 software (Artech Co. Ltd., Osaka, Japan). R version 3.6.3 (The R Foundation for Statistical Computing, Vienna, Austria) was used to perform the Steel–Dwass test for non-parametric multiple comparisons. Differences were considered significant if *P* < 0.05.

## Supplementary Information

Supplementary Information.

## Data Availability

The datasets generated during and/or analyzed during the current study are available from the corresponding author on reasonable request.
