# Supplementary material for: A novel highly quantitative and reproducible assay for the detection of anti-SARS-CoV-2 IgG and IgM antibodies
Source: Sci Rep. 2021 Mar 4;11:5198. doi: 10.1038/s41598-021-84387-3 (PMC7933429; doi:10.1038/s41598-021-84387-3)
Supplement: Supplementary file 1 — Supplementary Information. [file 41598_2021_84387_MOESM1_ESM.docx]

**A novel highly quantitative and reproducible assay for the detection of anti-SARS-CoV-2 IgG and IgM antibodies**

Kenta Noda, Kouki Matsuda, Shigehiro Yagishita, Kenji Maeda, Yutaro Akiyama, Junko Terada-Hirashima, Hiromichi Matsushita, Satoshi Iwata, Kazuto Yamashita, Yusuke Atarashi, Shunsuke Watanabe, Nobuyuki Ide, Tomokazu Yoshida, Norio Ohmagari, Hiroaki Mitsuya, Akinobu Hamada

**Corresponding Authors**

Akinobu Hamada, PhD.

Division of Molecular Pharmacology, National Cancer Center Research Institute, Tokyo, Japan

E-mail: akhamad@ncc.go.jp

Kenji Maeda, MD. PhD.

Department of Refractory Viral Infections, National Center for Global Health and Medicine (NCGM) Research Institute, Tokyo, Japan

E-mail: kmaeda@ri.ncgm.go.jp

Tomokazu Yoshida, Ph.D.

Central Research Laboratories, Sysmex Corporation, Hyogo, Japan

E-mail: Yoshida.Tomokazu@sysmex.co.jp

**Supplemental Table 1.** Evaluation of the sensitivity, specificity, and reproducibility of the novel assay

|  | N-IgG | | S-IgG | | N-IgM | | S-IgM | |
| --- | --- | --- | --- | --- | --- | --- | --- | --- |
| Sensitivity (%) | 100.0 | | 98.3 | | 71.7 | | 93.3 | |
| Specificity (%) | 99.8 | | 99.6 | | 83.4 | | 93.6 | |
| Reproducibility |  | |  | |  | |  | |
| Level^1^ | L | H | L | H | L | H | L | H |
| n | 10 | 10 | 10 | 10 | 10 | 10 | 10 | 10 |
| Mean (AU/mL) | 3.3 | 24.5 | 4.2 | 35.6 | 6.4 | 158.5 | 4.2 | 33.4 |
| SD | 0.0 | 0.5 | 0.1 | 0.9 | 0.1 | 2.6 | 0.1 | 0.4 |
| CV (%) | 1.4 | 2.1 | 3.3 | 2.5 | 2.1 | 1.7 | 1.2 | 1.2 |

^1^L and H indicate low and high concentration controls.

Abbreviations: CV, coefficient of variation; SD, standard deviation.

**Supplemental Table 2. Details of patient and sample information, and antibody titers**

| Patient ID | Age | Sex | Severity | Early stage sample collection | Convalescent sample collection | N-IgG [AU/mL] | | S-IgG [AU/mL] | | N-IgM [AU/mL] | | S-IgM [AU/mL] | |
| --- | --- | --- | --- | --- | --- | --- | --- | --- | --- | --- | --- | --- | --- |
|  |  |  |  | (Days from disease onset) | (Days from disease onset) | Early stage | Convalescent | Early stage | Convalescent | Early stage | Convalescent | Early stage | Convalescent |
| #1 | 65 | M | severe | 9 | 29 | 8.1 | 364.3 | 1.6 | 386.2 | 8.6 | 36.6 | 41.1 | 572.4 |
| #2 | 46 | F | moderate | 6 | 32 | 0.4 | 29.1 | 0.2 | 52.2 | 2.7 | 2.6 | 10.5 | 18.5 |
| #3 | 69 | F | moderate | NA | 35 | NA | 53.7 | NA | 59.2 | NA | 5.9 | NA | 54.7 |
| #4 | 62 | M | moderate | 12 | 38 | 27.7 | 228.7 | 15.8 | 404.7 | 19.3 | 13.8 | 140.6 | 133.0 |
| #5 | 53 | M | severe | 6 | 35 | 49.0 | 404.3 | 0.3 | 161.7 | 7.6 | 11.0 | 5.5 | 101.2 |
| #6 | 55 | M | moderate | 10 | 37 | 123.7 | 162.0 | 30.9 | 51.5 | 80.5 | 41.4 | 155.9 | 159.2 |
| #7 | 46 | M | moderate | NA | 42 | NA | 35.3 | NA | 37.8 | NA | 3.7 | NA | 21.4 |
| #8 | 53 | M | moderate | 7 | 48 | 3.0 | 377.3 | 0.1 | 420.6 | 9.9 | 23.6 | 121.7 | 77.4 |
| #9 | 61 | M | moderate | 13 | 66 | 128.1 | 99.2 | 17.7 | 233.6 | 16.3 | 4.6 | 89.1 | 105.1 |
| #10 | 23 | F | moderate | NA | 37 | NA | 40.1 | NA | 24.5 | NA | 36.0 | NA | 38.0 |
| #11 | 65 | F | moderate | 16 | 38 | 177.9 | 458.7 | 347.3 | 765.6 | 59.8 | 39.7 | 311.6 | 87.8 |
| #12 | 48 | M | moderate | NA | 47 | NA | 12.3 | NA | 1.5 | NA | 2.2 | NA | 1.1 |
| #13 | 31 | M | moderate | 8 | 33 | 13.3 | 301.3 | 0.2 | 60.6 | 25.3 | 116.1 | 9.2 | 178.8 |
| #14 | 53 | M | moderate | 5 | 84 | 0.1 | 104.7 | 0.2 | 50.9 | 1.5 | 15.6 | 0.4 | 195.8 |
| #15 | 46 | M | moderate | 8 | 50 | 0.1 | 282.3 | 0.1 | 91.7 | 1.2 | 10.5 | 1.9 | 10.6 |
| #16 | 44 | M | moderate | NA | 34 | NA | 316.6 | NA | 62.2 | NA | 14.0 | NA | 113.8 |
| #17 | 48 | F | moderate | 12 | 30 | 38.4 | 45.8 | 16.2 | 23.1 | 29.8 | 15.9 | 34.2 | 10.9 |
| Patient ID | Age | Sex | Severity | Early stage sample collection | Convalescent sample collection | N-IgG [AU/mL] | | S-IgG [AU/mL] | | N-IgM [AU/mL] | | S-IgM [AU/mL] | |
|  |  |  |  | (Days from disease onset) | (Days from disease onset) | Early stage | Convalescent | Early stage | Convalescent | Early stage | Convalescent | Early stage | Convalescent |
| #18 | 53 | M | moderate | 7 | 30 | 2.9 | 166.6 | 2.8 | 356.2 | 3.2 | 194.0 | 0.8 | 300.1 |
| #19 | 53 | M | severe | 3 | 95 | 0.1 | 28.7 | 0.1 | 21.6 | 0.9 | 3.2 | 0.3 | 3.8 |
| #20 | 51 | M | severe | 8 | 52 | 12.1 | 918.4 | 1.2 | 154.2 | 3.6 | 23.4 | 7.0 | 54.6 |
| #21 | 41 | M | moderate | 1 | 86 | 0.1 | 51.4 | 0.1 | 62.2 | 1.0 | 5.5 | 0.2 | 12.7 |
| #22 | 46 | M | moderate | 5 | 65 | 24.1 | 78.4 | 9.9 | 183.4 | 9.3 | 8.6 | 92.8 | 23.8 |
| #23 | 25 | M | moderate | 5 | 62 | 0.2 | 20.5 | 0.2 | 15.6 | 2.6 | 4.0 | 0.9 | 4.5 |
| #24 | 49 | M | moderate | 11 | 63 | 72.8 | 71.0 | 23.4 | 94.4 | 4.6 | 1.9 | 71.7 | 15.4 |
| #25 | 47 | F | moderate | 3 | 56 | 0.2 | 14.5 | 0.2 | 10.7 | 11.9 | 12.0 | 8.5 | 11.4 |
| #26 | 34 | F | moderate | 10 | 42 | 58.0 | 101.9 | 10.4 | 51.3 | 14.1 | 5.5 | 81.9 | 28.0 |
| #27 | 37 | M | severe | 10 | 58 | 17.1 | 75.2 | 3.6 | 380.8 | 8.8 | 21.2 | 50.6 | 111.7 |
| #28 | 67 | M | severe | 10 | 56 | 499.0 | 266.8 | 2.4 | 302.8 | 264.5 | 51.1 | 38.6 | 44.8 |
| #29 | 38 | M | moderate | NA | 61 | NA | 101.6 | NA | 38.4 | NA | 3.7 | NA | 47.5 |
| #30 | 45 | M | moderate | NA | 56 | NA | 97.0 | NA | 28.2 | NA | 4.8 | NA | 69.0 |
| #31 | 43 | M | moderate | NA | 68 | NA | 137.3 | NA | 150.0 | NA | 5.6 | NA | 15.8 |
| #32 | 68 | M | moderate | NA | 25 | NA | 217.2 | NA | 274.0 | NA | 131.4 | NA | 287.1 |
| #33 | 38 | M | moderate | NA | 65 | NA | 179.0 | NA | 200.5 | NA | 14.1 | NA | 10.5 |
| #34 | 78 | M | critical | 13 | 32 | 2.6 | 140.6 | 1.4 | 216.4 | 10.2 | 23.7 | 50.4 | 196.1 |
| #35 | 67 | M | critical | 18 | 34 | 106.9 | 159.2 | 6.3 | 112.5 | 18.4 | 16.2 | 461.7 | 778.7 |
| Patient ID | Age | Sex | Severity | Early stage sample collection | Convalescent sample collection | N-IgG [AU/mL] | | S-IgG [AU/mL] | | N-IgM [AU/mL] | | S-IgM [AU/mL] | |
|  |  |  |  | (Days from disease onset) | (Days from disease onset) | Early stage | Convalescent | Early stage | Convalescent | Early stage | Convalescent | Early stage | Convalescent |
| #36 | 36 | M | critical | 7 | 36 | 304.9 | 270.4 | 164.7 | 127.3 | 58.4 | 16.7 | 125.8 | 19.1 |
| #37 | 79 | F | severe | 14 | 32 | 241.0 | 139.2 | 337.1 | 242.6 | 62.8 | 26.5 | 487.9 | 171.8 |
| #38 | 34 | M | severe | 6 | 57 | 0.8 | 127.0 | 0.2 | 117.5 | 5.9 | 127.4 | 3.1 | 98.0 |
| #39 | 81 | M | severe | 9 | 31 | 0.3 | 105.6 | 0.3 | 279.1 | 3.4 | 19.1 | 2.8 | 162.0 |
| #40 | 57 | M | critical | 12 | 36 | 0.7 | 106.8 | 0.1 | 433.8 | 7.0 | 43.9 | 15.4 | 72.7 |
| #41 | 71 | M | moderate | 25 | 36 | 3.8 | 85.5 | 2.1 | 184.9 | 11.0 | 35.8 | 111.1 | 301.2 |
| #42 | 53 | M | critical | 11 | 25 | 51.5 | 319.2 | 84.4 | 493.4 | 15.2 | 18.6 | 252.0 | 550.5 |
| #43 | 34 | F | moderate | 8 | 40 | 0.4 | 164.3 | 0.3 | 119.0 | 17.8 | 55.3 | 9.7 | 378.1 |
| #44 | 47 | M | severe | 10 | 34 | 6.6 | 127.9 | 2.2 | 488.5 | 5.7 | 26.7 | 30.0 | 170.9 |
| #45 | 29 | F | moderate | 8 | 30 | 0.9 | 100.9 | 0.7 | 103.9 | 3.6 | 74.2 | 8.4 | 41.8 |
| #46 | 71 | M | severe | 9 | 38 | 2.2 | 127.6 | 0.4 | 97.1 | 2.1 | 6.7 | 9.3 | 30.9 |
| #47 | 44 | M | moderate | 5 | 28 | 0.5 | 254.1 | 0.7 | 309.3 | 2.1 | 36.5 | 3.6 | 500.1 |
| #48 | 50 | M | severe | 13 | 24 | 250.1 | 309.1 | 185.4 | 489.4 | 118.1 | 44.9 | 304.9 | 167.7 |
| #49 | 3 | M | moderate | 8 | 23 | 0.4 | 96.3 | 3.4 | 187.1 | 2.6 | 5.5 | 83.7 | 28.9 |
| #50 | 48 | M | severe | 7 | 37 | 1.4 | 214.7 | 0.5 | 361.2 | 8.7 | 15.7 | 11.8 | 85.9 |
| #51 | 79 | M | severe | 9 | 26 | 5.2 | 268.2 | 0.4 | 186.5 | 1.8 | 10.0 | 2.1 | 86.2 |
| #52 | 57 | M | severe | 10 | 39 | 4.7 | 127.1 | 0.2 | 234.8 | 129.8 | 419.9 | 7.2 | 65.8 |
| #53 | 71 | M | moderate | 13 | 35 | 188.8 | 283.2 | 90.0 | 720.4 | 42.8 | 51.5 | 335.6 | 148.2 |
| Patient ID | Age | Sex | Severity | Early stage sample collection | Convalescent sample collection | N-IgG [AU/mL] | | S-IgG [AU/mL] | | N-IgM [AU/mL] | | S-IgM [AU/mL] | |
|  |  |  |  | (Days from disease onset) | (Days from disease onset) | Early stage | Convalescent | Early stage | Convalescent | Early stage | Convalescent | Early stage | Convalescent |
| #54 | 87 | M | moderate | 9 | 20 | 76.8 | 193.4 | 10.7 | 236.9 | 30.5 | 64.3 | 118.6 | 542.9 |
| #55 | 22 | M | moderate | 5 | 33 | 87.1 | 108.8 | 1.1 | 24.1 | 12.5 | 5.6 | 9.8 | 7.3 |
| #56 | 45 | M | severe | 11 | 30 | 2.5 | 270.6 | 0.3 | 258.0 | 49.6 | 259.8 | 12.3 | 316.0 |
| #57 | 74 | M | severe | 13 | 26 | 88.7 | 358.1 | 5.2 | 149.1 | 129.5 | 1685.7 | 34.2 | 68.0 |
| #58 | 70 | M | severe | 12 | 34 | 107.1 | 720.0 | 0.9 | 513.1 | 5.7 | 40.9 | 4.5 | 28.7 |
| #59 | 85 | F | severe | 6 | 22 | 0.1 | 176.1 | 0.2 | 156.9 | 1.3 | 14.8 | 0.7 | 140.6 |
| #60 | 85 | F | moderate | 6 | 28 | 0.3 | 88.2 | 0.3 | 101.1 | 2.9 | 387.8 | 1.9 | 99.0 |

*NA: not applicable


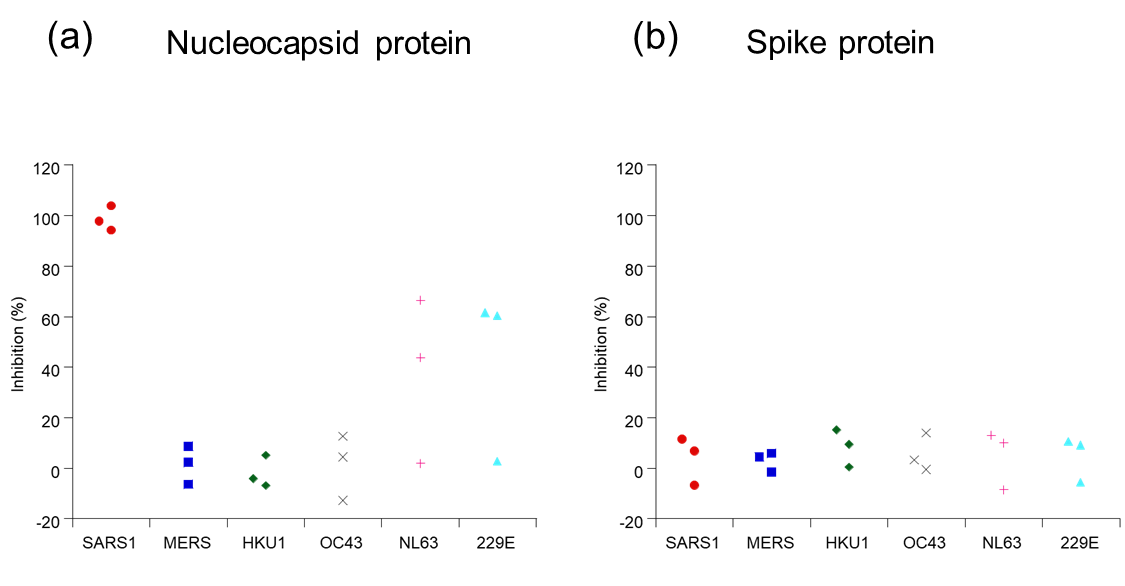


**Supplemental Figure 1:** Specificity of the SARS-CoV-2 antibody test. The inhibition rate with SARS-CoV-2 antigen was set at 100%, and crossover by other human coronavirus-derived antigens was assessed. Inhibition rate of nucleocapsid (a) and spike (b) proteins is shown. Common human coronaviruses: 229E and NL63 (alpha coronavirus), HKU1 and OC43 (beta coronavirus). MERS, Middle East respiratory syndrome; SARS1, severe acute respiratory syndrome.


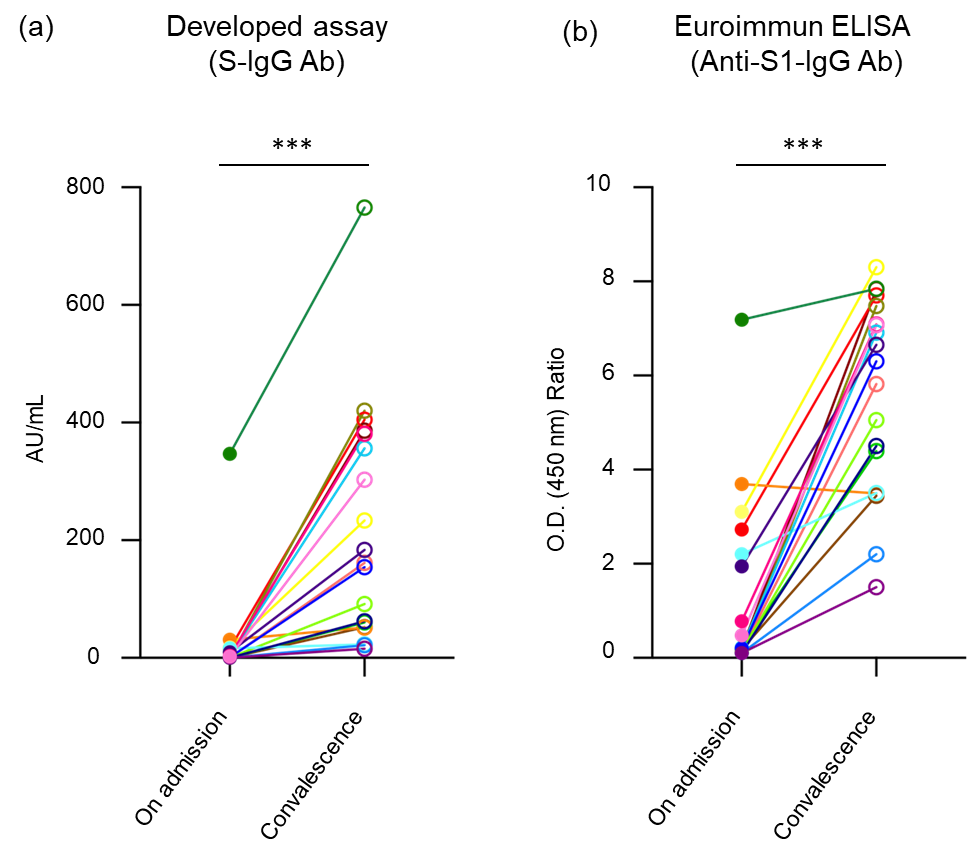


**Supplemental Figure 2:** Comparison between the high-sensitivity chemiluminescence enzyme immunoassay (HISCL) analysis system and a commercially available enzyme-linked immunosorbent assay (ELISA). The amounts of anti-SARS-CoV-2-IgG in the serum of 19 patients were measured with a commercial ELISA kit [Anti-SARS-CoV-2 ELISA (IgG) (EUROIMMUN AG, Luebeck, Germany)], and the data were compared with those of the novel HISCL analysis system. Values for each patient on admission and at a convalescence time point are connected by a line. Significant differences between SARS-CoV-2-IgG levels were determined upon using both assays (at the time of admission as well as convalescence) were observed. ****P* > 0.001.


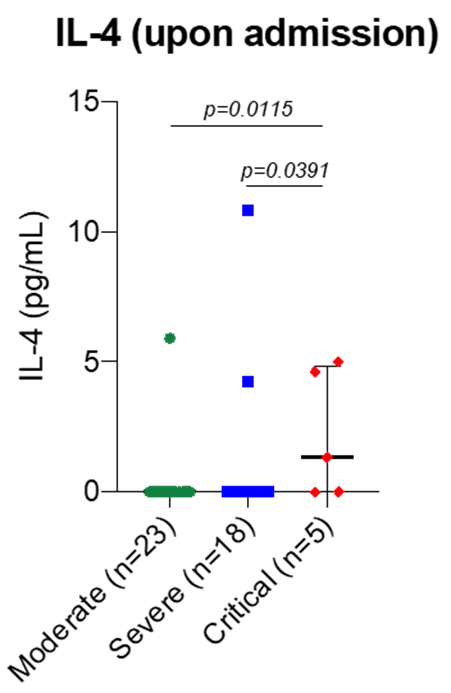


**Supplemental Figure 3: Relationship between plasma IL-4 protein level and the severity status of patients.**

The plasma IL-4 level in the blood of each patient on admission was measured and analyzed for the relationship with the severity status of patient.
